# Supplementary material for: Interaction between Pharmaceutical Drugs and Polymer-Coated Fe3O4 Magnetic Nanoparticles with Langmuir Monolayers as Cellular Membrane Models
Source: Pharmaceutics. 2023 Jan 17;15(2):311. doi: 10.3390/pharmaceutics15020311 (PMC9961141; doi:10.3390/pharmaceutics15020311)
Supplement: Supplementary file 1 [file pharmaceutics-15-00311-s001.zip › pharmaceutics-2109372-supplementary.pdf]

Interaction between Pharmaceutical Drugs and Polymer-Coated Fe_3O_4 Magnetic Nanoparticles with Langmuir Monolayers as Cellular Membrane Models

Sara Natalia Moya Betancourt [1,2], Candelaria Inés Cámara [1,2] and Julieta Soledad Riva [1,2,*]

[1] Consejo Nacional de Investigaciones Científicas y Técnicas (CONICET). Instituto de Fisicoquímica y Química Inorgánica de Córdoba (INFIQC), 5000 Córdoba, Argentina

[2] Departamento de Fisicoquímica. Facultad de Ciencias Químicas, Universidad Nacional de Córdoba. Haya de la Torre y Medina Allende, Ciudad Universitaria, 5000 Córdoba, Argentina

* Correspondence: Julieta.riva@unc.edu.ar

Supporting Information

S1. Results

S1.1. Elastic modulus for MNPs:DSPA mixtures monolayer containing $HTFPZ^+$ or $DCFN^-$ in the subphase

The changes in Cs^{-1} of DSPA monolayers at different MNPs volume for mixed monolayer in presence of $HTFPZ^+$ or $DCFN^-$ in the subphase are shown in Figures S1 and S2, respectively. In presence of increasing amount of MNPs, the of Cs^{-1} values became progressively smaller. As the collapse pressure decrease in the isotherms, also the surface pressure of Cs^{-1} decrease.

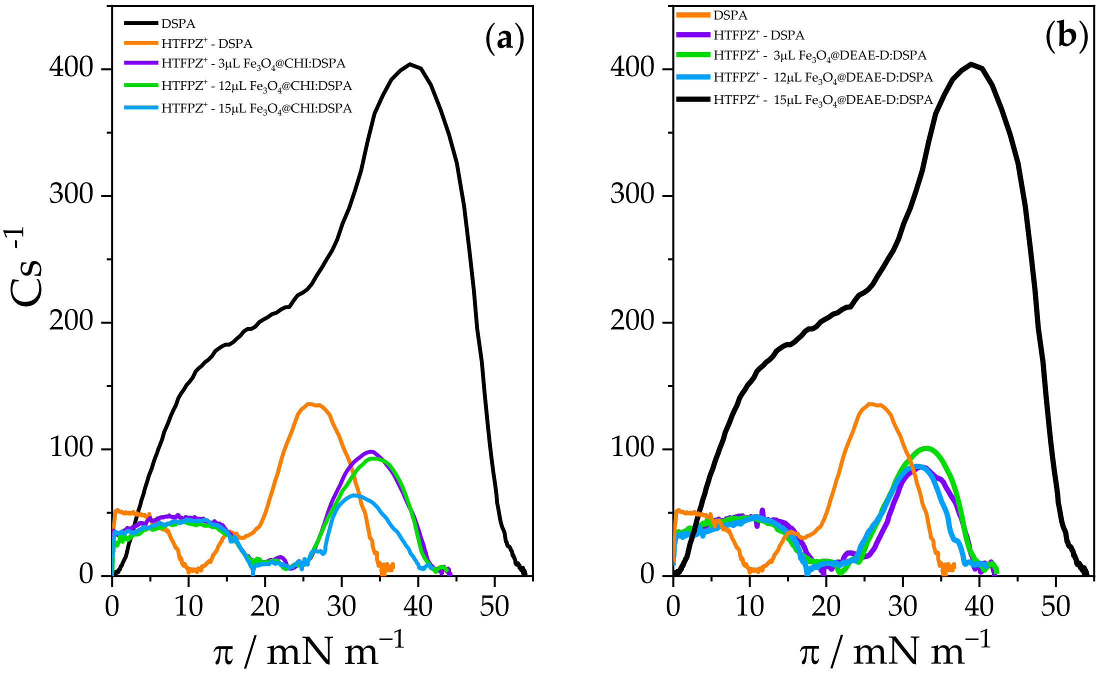

Figure S1. CS^{-1} as a function of pressure (π) for (**a**) Fe_3O_4@CHI:DSPA and (**b**) Fe_3O_4@DEAE-D:DSPA, calculated from π-A isotherms using Equation (1). Subphase composition: $HTFPZ^+$ in LiCl 10 mM and LiCl 10 mM for black line.

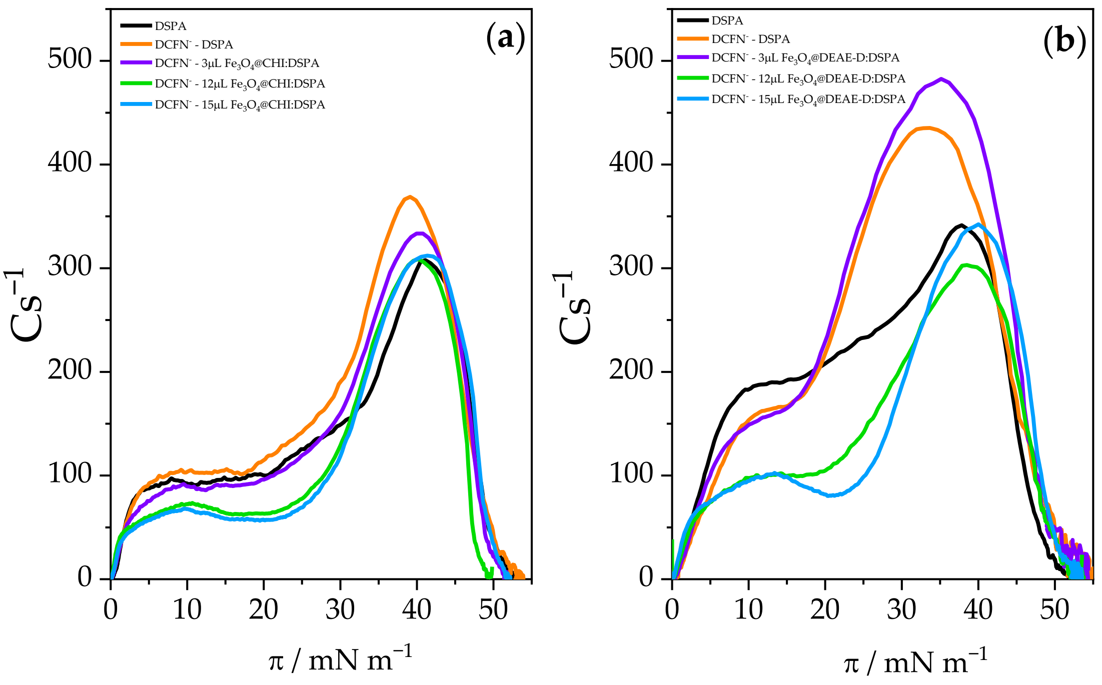

Figure S2. CS^{-1} as a function of pressure (π) for (**a**) Fe_3O_4@CHI:DSPA and (**b**) Fe_3O_4@DEAE-D:DSPA, calculated from π-A isotherms using Eq. (1). Subphase composition: DCFN− in LiCl 10 mM and LiCl 10 mM for black line.
